# Effect of Drying Protocols on the Bond Strength of Bioceramic, MTA and Resin-based Sealer Obturated Teeth

**DOI:** 10.5005/jp-journals-10005-1589

**Published:** 2019

**Authors:** Nishant Khurana, Hemant R Chourasia, Gautam Singh, Khusboo Mansoori, Adamya S Nigam, Babita Jangra

**Affiliations:** 1 Department of Conservative Dentistry and Endodontics, Peoples College of Dental Sciences and Research Centre, Bhopal, Madhya Pradesh, India; 2 Department of Endodontics, College of Dentistry, Jazan, Saudi Arabia; 3 Department of Conservative Dental Sciences, IBN Sina National College of Medical Studies, Jeddah, Saudi Arabia; 4 Department of Conservative Dentistry and Endodontics, Guru Govind Singh College of Dental Sciences and Research Centre, Bhuranpur, Madhya Pradesh, India; 5 Dr. Nigam Dental Clinic, Vidisha, Madhya Pradesh, India; 6 Department of Pedodontics and Preventive Dentistry, Maulana Azad Institute of Dental Sciences, New Delhi, India

**Keywords:** Bioceramic-based sealer, Bond strength, Isopropyl alcohol, MTA-based sealer, Push-out test, Resin-based sealer

## Abstract

**Aim:**

The aim of this study was to evaluate the bond strength and thereafter analyze the mode of failure of the three sealers applied to smear free radicular dentine with final drying using 70% isopropyl alcohol and paper points.

**Materials and methods:**

A total of sixty root canals were prepared and then segregated into two groups (*n* = 30) as per the drying protocol, namely paper points or 70% isopropyl alcohol. Then, these roots were divided into three sub-groups (*n* = 10) with respective sealers and obturation materials, namely AH Plus and gutta-percha (AH/GP), EndoSequence BC and gutta-percha (EBC/GP), and MTA Fillapex and gutta-percha (MFP/GP). The roots were then sectioned from each third, and the push-out test was performed. Failure modes were examined under a stereomicroscope. Data were statistically analyzed by 2-way analysis of variance *post hoc* Tukey tests with a significant level of 5%.

**Results:**

Overall canals dried with isopropyl alcohol showed higher bond strength values than paper point (*p* < 0.05). The AH/GP group showed lower bond strength than EBC/GP (*p* < 0.05) but higher than MFP/GP (*p* < 0.05). The most frequent type of failure was cohesive in the AH/GP group and adhesive in the EBC/GP group whereas MFP/GP had almost similar adhesive and cohesive failures.

**Conclusion:**

Seventy percent isopropyl alcohol drying improved the bond strength of the root canal sealers with the dentinal tubules better than the ideal paper point drying.

**How to cite this article:**

Khurana N, Chourasia HR, *et al.* Effect of Drying Protocols on the Bond Strength of Bioceramic, MTA and Resin-based Sealer Obturated Teeth. Int J Clin Pediatr Dent 2019;12(1):33–36.

## INTRODUCTION

An effective endodontic seal blocks communication between the root canal system and surrounding periapical tissues.^1^ Ceremoniously, a root canal filling consists of a core, i.e., gutta-percha (GP), which is adapted to the canal walls with an endodontic sealer thereby filling the voids and gaps. Endodontic sealers with GP make an optimal seal and an improper seal often leads to endodontic failure.^2^ Sealer adhesion to dentin may be affected by the moisture condition of the root canals; henceforth, drying the root canal before obturation increases the sealers adherence to the dentinal walls and the filling material.^1–4^

Clinicians have varied perceptions of moisture as manufacturers recommend that the canals should be maintained in a moist state to benefit from the hydrophilic properties of their sealer. But, an exact degree of moisture and method to achieve is never mentioned, which is ideal for their products.^5^ Considering that no clear instructions have been provided for achieving an ideal degree of residual moisture, various chemicals including alcohol at different concentrations have been tested to improve dentinal wettability.^3^

The epoxy resin-based sealers, such as AH Plus (Dentsply, Germany), are frequently used because of their reduced solubility, long-term dimensional stability, and adequate micro retention to dentin.^3^ A mineral trioxide aggregate (MTA)-based root canal sealer, MTA Fillapex (Angelus, Brazil), has been introduced, which contains salicylate resin with MTA and shows suitable physical properties to be used as an endodontic sealer.^6,7^ The more recently introduced bioceramic-based sealer, EndoSequence BC Sealer (Brasseller, USA)is an aluminum-free material with a calcium silicate composition, which is insoluble and radiopaque.^8^

Moisture may prevent sealer setting by increasing or reducing its working or setting time, and sealer penetration as well bond strength.^6^ Therefore, this study aimed to compare the push-out bond strength and failure analysis of an epoxy-based root canal sealer AH Plus, a bioceramic-based root canal sealer EndoSequence BC, and a calcium silicate-based root canal sealer MTA Fillapex, which are applied to smear free root dentin using paper points and 70% isopropyl alcohol drying.

The null hypothesis tested was that different drying protocols would not affect the bond strength of any of the tested endodontic sealers.

## MATERIALS AND METHODS

Sixty (*n* = 60) intact single rooted, single canal mandibular premolars with closed apices were collected from the tooth bank. The specimens were decoronated by transversely sectioning the roots at 15 mm, standardized using a digital Vernier Calliper from the apex with a double-faced diamond disc at a low speed with an air/water spray coolant.

The canals were prepared using a crown-down technique with rotary Protaper up to size F5, flushed with 2 mL of 5% sodium hypochlorite between each file size, and delivered using a syringe with a 30-G side vented needle placed 1 mm short of the working length (WL). After preparation, the canals were irrigated with 5 mL of 17% EDTA (pH = 7.7) for 5 minutes followed by a final 5 minutes 5 mL rinse with bidistilled water.

These specimens were then randomly assigned to three experimental groups (*n* = 20) according to the sealer used and sub divided into two sub-experimental groups (*n* = 10) according to the drying protocol.

**Fig. 1:**
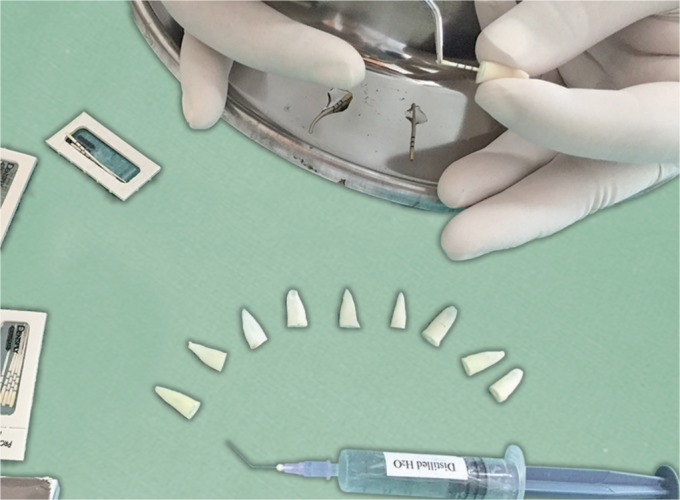
Paper point drying

In sub group I, the canals were blot dried using size F5 paper points until complete dryness of the last point was confirmed visually (Fig. 1).

**Fig. 2:**
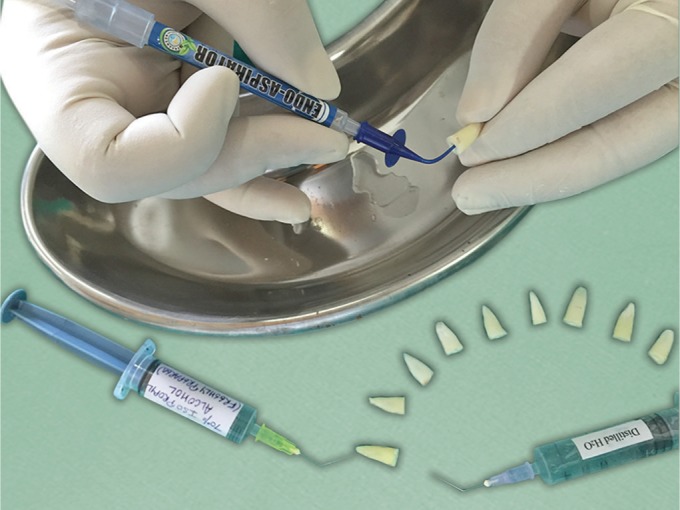
Isopropyl alcohol drying

In sub group II, after the removal of excess normal saline with size F5 paper points, as in sub group I, the canals were filled with 70% isopropyl alcohol (freshly prepared) using a syringe with a 30-G blunt-tip needle carried to the WL. The alcohol was left in the canal for 5 seconds and immediately aspirated using an Endo-Aspirator (Cerkamed, Polska) with a size 0.014 capillary tip in a low vacuum with a gentle up-and-down motion for 5 seconds (Fig. 2).

## SPECIMENS WERE FURTHER ASSIGNED TO GROUPS

Group I: GP with AH Plus (AH/GP) (Ia + Ib = *n* = 20)

Group II: GP with EndoSequence BC (EBC/GP) (IIa + IIb = *n* = 20)

Group III: GP with MTA Fillapex (MFP/GP) (IIIa + IIIb = *n* = 20).

The sealers were prepared according to the manufacturer's recommendations and introduced into the canal with a pre-fitted size F5 cone to the full WL, and lateral compaction using accessory GP cones was conducted. A heated Hand Plugger no. #3-4 instrument was used to cut the coronal surplus and vertically compact the same. The roots were radiographically verified and if any discrepancy was found the sample was replaced. Then, the samples were stored (37 °C and 95% humidity) for 7 days to allow complete setting of the sealers.

### Sectioning of Samples

Each root third (coronal, middle, and apical) was sectioned perpendicularly to its long axis into one 2 mm-thick slice using a double faced diamond disc under water stream. Thus, three slices were obtained from each specimen, with a total of 60 sections per group. Each slice was marked on its apical side with an indelible marker. The slice obtained from each root canal third was submitted to the push-out test in a universal testing machine, with tip diameters of 0.5 mm, 0.8 mm, and 1.0 mm used for the apical, middle, and coronal sections, respectively, until bond failure.

The apical surface displaying the ink dot was placed facing the punch tip, ensuring that loading forces were introduced from an apical to coronal direction; thus, avoiding any limitation to the material movement. This method ensured the alignment of the specimen in an accurate and reproducible manner, maintained the shaft centralized, and avoided its contact with the dentin when the material was pushed and dislodged from the canal wall.

Bond strength data were converted to MPa by dividing the load (in kN) by the adhesion area of the filling material in square millimeters. The adhesion area was calculated as the lateral surface area of a truncated cone using the formula *π*(*R* + *r*)[*h*^2^ + (*R* − *r*)^2^]^0.5^, where *π* is the constant 3.14, *R* is the mean radius of the coronal canal, *r* is the mean radius of the apical canal, and *h* is the thickness of the slice. The widest and narrowest diameters of the filling material and the thickness of the slice were individually measured using a digital calliper with 0.001 mm accuracy.

**Figs 3A to C:**
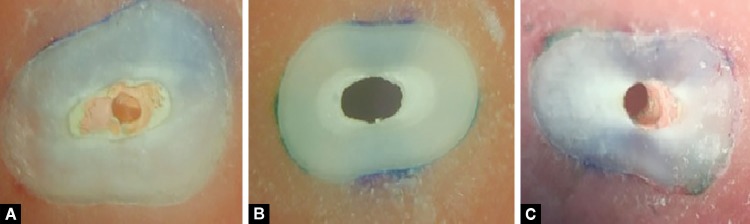
(A) Cohesive failure; (B) Adhesive failure; (C) Mixed failure (stereomicroscopic image under 40× magnification)

The failure mode of each debonded specimen after push-out test was assessed with a stereomicroscope at a magnification of 40×. Failures are classified as follows:

- Adhesive failure between dentin and sealer (no sealer visible on dentin walls)
- Cohesive failure in sealer (dentin walls totally covered with sealer) mixed when both adhesive and cohesive failures could be observed. Figure 3 shows a composite picture of stereomicroscopic images where the three failure modes can be visualized accordingly.

A comparative evaluation was made and assessed according to the data obtained.

### Statistical Analysis

The samples tested were analyzed by two-way analysis of variance. *Post hoc* Tukey test was used to determine whether a significant two-factor interaction existed between the experimental groups, i.e., in between the sealer and the drying protocols. The mode of failure was analyzed by the Chi square test. Statistical comparison within and between the experimental groups was performed by using SPSS v21.0 (IBM Corporation, Armonk, New York, USA) with the significance level set at 5%.

## RESULT

The mean and standard deviation of push-out bond strength of the experimental groups at each third are summarized in Table 1. Bond strength values are significantly affected within each section in all the sealer groups by the isopropyl drying protocol (*p* < 0.05).

The EBC/GP group displayed a significantly higher bond strength than the other group (*p* < 0.05). A statistical ranking for bond strength values was EBC/GP > AH/GP > MFP/GP groups. Also the strength was enhanced by the isopropyl drying group.

Analysis of bond failure after the push-out test as in Table 2 showed that AH Plus had major cohesive failures while EndoSequence BC had major adhesive failure whereas MTA Fillapex showed adhesive and cohesive failure equally.

## DISCUSSION

Since GP has no adhesion to the dentin surface, the sealer should have adequate flow for filling gaps between GP cones and the canal walls and impart bond strength to root dentin. In addition, a sealer should have adhesive strength and also have cohesive strength to hold the obturation together. Endodontic sealers are responsible for sealing the root canal system, entombing remaining bacteria and filling irregularities in the prepared canal system.^9^

Non-resin-based endodontic sealers may also offer adhesive properties comparable to those of resin-based ones. An ideal root canal sealer should also adhere to both dentin and the core filling material.^5^

In the present study, three sealers have been used: a resin-based sealer (AH Plus), a bioceramic root canal sealer (EndoSequence BC), and a MTA-based root canal sealer (MTA Fillapex) with two drying protocols, i.e., paper point drying and 70% isopropyl alcohol. The result showed that 70% isopropyl alcohol (C_3_H_7_OH) revealed higher bond strength; therefore, the null hypothesis was rejected.

**Table 1:** Push-out bond strength (MPa) recorded for different sealers and canal regions

*Sealers*	*Mean ± SD of push-out bond strength (MPa)*							
	*Coronal*			*Middle*			*Apical*	
	*Paper point drying*	*70% isopropyl alcohol drying*		*Paper point drying*	*70% isopropyl alcohol drying*		*Paper point drying*	*70% isopropyl alcohol drying*
AH Plus	4.29 ± 0.06	**4.66 ± 0.06**		3.32 ± 0.07	**4.24 ± 0.06**		2.27 ± 0.07	**2.80 ± 0.05**
EndoSequence BC	4.63 ± 0.06	**4.97 ± 0.06**		3.65 ± 0.07	**4.59 ± 0.06**		2.59 ± 0.06	**3.09 ± 0.06**
MTA Fillapex	1.53 ± 0.06	**1.98 ± 0.05**		0.95 ± 0.06	**1.51 ± 0.06**		0.65 ± 0.06	**1.07 ± 0.06**

**Table 2:** Failure mode distribution at each root third of sealers

*Root section*	*Failure mode*	*AH Plus*			*EndoSequence BC*			*MTA Fillapex*	
		*Paper point *n* (%)*	*70% isopropyl alcohol drying *n* (%)*		*Paper point *n* (%)*	*70% isopropyl alcohol drying *n* (%)*		*Paper point *n* (%)*	*70% isopropyl alcohol drying *n* (%)*
Coronal	Adhesive	00 (0.00)	00 (0.00)		**07 (70.00)**	**06 (60.00)**		**04 (40.00)**	**05 (50.00)**
	Cohesive	**08 (80.00)**	**09 (90.00)**		02 (20.00)	02 (20.00)		04 (40.00)	04 (40.00)
	Mixed	02 (20.00)	01 (10.00)		01 (10.00)	02 (20.00)		02 (20.00)	01 (10.00)
Middle	Adhesive	00 (0.00)	01 (10.00)		**06 (60.00)**	**07 (70.00)**		04 (40.00)	04 (40.00)
	Cohesive	**08 (80.00)**	**09 (90.00)**		02 (20.00)	02 (20.00)		**05 (50.00)**	**05 (50.00)**
	Mixed	02 (20.00)	00 (0.00)		02 (20.00)	01 (10.00)		01 (10.00)	01 (10.00)
Apical	Adhesive	00 (0.00)	00 (0.00)		**06 (60.00)**	**06 (60.00)**		**04 (40.00)**	**05 (50.00)**
	Cohesive	**07 (70.00)**	**09 (90.00)**		00 (0.00)	03 (30.00)		05 (50.00)	04 (40.00)
	Mixed	03 (30.00)	01 (10.00)		04 (40.00)	01 (10.00)		01 (10.00)	01 (10.00)

In endodontic research, epoxy resin-based sealers, such as AH Plus, are frequently used but its sealing ability remains controversial partly because AH Plus does not bond to gutta-percha.^3^

MTA based sealers that have been introduced like MTA-Fillapex, which is present in two-paste form has a part from MTA; its chemical composition contains resins, bismuth oxide, silica nanoparticles, and dyes. This sealer has high sealing ability, radiopacity, low solubility as well as low setting expansion, bactericidal effect, and biocompatibility.^10^

EndoSequence BC Sealer is a new hydrophilic calcium silicate-based sealer that can be used for filling root canals with or without GP.^11^ This bioceramic sealer (BCS) is an insoluble, radiopaque, and aluminum-free material with a calcium silicate composition, which requires the presence of water to set and harden. Additionally, it has a similar composition to white MTA and has shown excellent physical properties and antimicrobial activity.^5^

Considering that no clear instructions have been provided for achieving such an ideal degree of residual moisture,^12^ various chemicals, including alcohol at different concentrations,^5,13,14^ have been tested to improve dentinal wettability. Recent studies have shown that excessive desiccation may remove the water residing in the dentinal tubules, which may in turn hamper the effectiveness of hydrophilic sealers and adhesion.^5,12^ The differences in the dentinal tubule density and the limited accessibility of the solutions to the most apical portions of the canal may explain significant differences in the results observed among the canal thirds in some groups.^3^

It has been suggested that the push-out test provides a better evaluation of bonding strength than the conventional shear test because using the push-out test, fracture occurs parallel to the dentine-bonding interface, which makes it a true shear test for parallel-sided samples.^15^

Most of the manufacturers recommend drying of the root canal system before application of the root canal sealer to enhance the sealer penetration into the radicular dentin and optimize the sealing ability of the sealer. The use of paper points have been advocated for the above system, which uses the principle of direct contact and capillary action for absorbing and adsorbing the water but some of the water may be left behind in the root canal system because of its known complex anatomies, which in turn may form an in displaceable physical barrier for the complete penetration of various endodontic sealers.

After the push-out bond strength evaluations, the samples were observed under a stereomicroscope, which revealed that AH/GP (group I) showed about 70–90% cohesive failures and 10–30% mixed failures. EBC/GP (group II) showed about 60–70% adhesive failures, 0–30% cohesive failures, and 10–40% mixed failures and MFP/GP (group III) showed about 40–50% adhesive failures, 40–50 % cohesive failure, and 10–20% mixed failure at all thirds.

Thus, within the limitation of the study, it can be concluded that the push-out bond strength of EndoSequence BC sealer > AH Plus > MTA Fillapex at all thirds of the root canal as well as in both drying protocols was observed. The present study was performed *in vitro*; it is suggested that it would be effective *in vivo* and in multirooted teeth where effective drying is difficult to achieve. It can be recommended in pediatric dentistry to achieve effective drying while obturating permanent teeth or primary teeth where succedaneous tooth is missing.

## CONCLUSION

In this study, the effectiveness of two different drying protocols was checked; first, the conventional paper point drying and second, the 70% freshly prepared isopropyl alcohol drying protocol, concluding that the 70% isopropyl alcohol drying protocol significantly increases the bond strength at each third and with all the sealers.
